# Association of the C-1019G (rs6295) Polymorphism of the *5-HT1A* Receptor Gene, Sociodemographic Variables, and Clinical Characteristics with Depressive Symptoms in a Mexican Population

**DOI:** 10.3390/diseases14070255

**Published:** 2026-07-14

**Authors:** Margarita Hernández-Mixteco, Olga Lidia Valenzuela, Hanna Belen Baranda-Jiménez, Ricardo Jiovanni Soria-Herrera, Manuel González-del Carmen, Paola Castillo-Juárez, Karla Gabriela Domínguez-González, Oscar Villavicencio-Carrisoza, Moises León-Juárez, Addy Cecilia Helguera-Repetto, Victoria Campos-Peña, Eliud Alfredo Garcia-Montalvo, Jorge Francisco Cerna-Cortés

**Affiliations:** 1 Departamento de Microbiología, Escuela Nacional de Ciencias Biológicas, Instituto Politécnico Nacional, Ciudad de México 11340, Mexico; hbarandaj1700@alumno.ipn.mx (H.B.B.-J.); pcastilloj@ipn.mx (P.C.-J.); 2 Facultad de Ciencias Químicas, Universidad Veracruzana, Orizaba 94340, Veracruz, Mexico; ovalenzuela@uv.mx (O.L.V.); elagarcia@uv.mx (E.A.G.-M.); 3 Facultad de Medicina, Universidad Veracruzana, Mendoza 94740, Veracruz, Mexico; manugonzalez@uv.mx; 4 Facultad de Químico Farmacobiología, Universidad Michoacana de San Nicolás de Hidalgo, Morelia 58240, Michoacán, Mexico; ricardo.soria@umich.mx; 5 Instituto de Investigaciones en Ciencias de la Tierra, Universidad Michoacana de San Nicolás de Hidalgo, Morelia 58040, Michoacán, Mexico; karla.dominguez@umich.mx; 6 Departamento de Medicina Traslacional, Instituto Nacional de Perinatología Isidro Espinosa de los Reyes, Ciudad de México 11000, Mexico; oscar.villavicencio@inper.gob.mx; 7 Departamento de Inmunobioquímica, Instituto Nacional de Perinatología Isidro Espinosa de los Reyes, Ciudad de México 11000, Mexico; moises.leon@inper.edu.mx (M.L.-J.); addy.helguera@inper.gob.mx (A.C.H.-R.); 8 Laboratorio Experimental de Enfermedades Neurodegenerativas, Instituto Nacional de Neurología y Neurocirugía, Manuel Velasco Suárez, Ciudad de México 14269, Mexico; neurovcp@ymail.com

**Keywords:** depressive symptoms, C-1019G polymorphism, *5-HT1A* receptor gene, sociodemographic variables, clinical characteristics, Mexican population, risk factors

## Abstract

Background/Objectives: Depression ranks among the most prevalent mental health disorders worldwide. Numerous studies have linked the C-1019G (rs6295) polymorphism in the serotonin 1A (*5-HT1A*) receptor gene to increased susceptibility to depression. In this study, sociodemographic variables and clinical characteristics were integrated with targeted genotyping of the C-1019G polymorphism to identify risk factors associated with depressive symptoms in a Mexican cohort. Methods: Sociodemographic and clinical data were collected from 257 volunteers (202 healthy controls, 55 with depressive symptoms). Genotyping for C-1019G was performed using polymerase chain reaction–restriction fragment length polymorphism (PCR-RFLP). Sociodemographic variables, clinical characteristics, and genotype frequencies were analyzed relative to Beck Depression Inventory II (BDI-II) scores. Results: BDI scores were significantly lower among males, participants aged < 40 years, unmarried individuals, and those with a high school education (*p* < 0.05). Females were three times more likely to exhibit depressive symptoms than males (*p* = 0.004; OR = 3.08). Compared with participants aged < 40 years, those aged 40–65 and >65 years were two (*p* = 0.026; OR = 2.19) and five times (*p* < 0.001; OR = 5.71) more likely to report depressive symptoms, respectively. Participants with type 2 diabetes mellitus had twice the odds of depressive symptoms (*p* = 0.025; OR = 2.38). Odds increased twelvefold among those with diabetes plus arterial hypertension (*p* < 0.001; OR = 12.25). Among males, the C/C genotype was observed exclusively in controls (*p* = 0.010). Furthermore, the genotypic distribution of this study population differed from that of European and Asian populations. Conclusions: These findings advance the understanding of depressive symptoms’ multifactorial etiology and may inform targeted screening and prevention strategies.

## 1. Introduction

Depressive disorder represents a major global public health challenge. Globally, the prevalence of depressive disorders increased by 88.52% from 1990 to 2021, affecting hundreds of millions of people and producing substantial disability and lost years of healthy life across regions [1]. Regional data show a pooled lifetime prevalence of depressive disorder of 12.58% in Latin America [2]. In the Mexican population, the National Health and Nutrition Survey (ENSANUT) estimated that, overall, 16.7% of adults (aged ≥ 20 years) screened positive for depressive symptoms, being higher in older adults (≥60 years). Additionally, 45.8% of adolescents reported at least one depressive symptom in the week before the survey [3].

Risk factors for the onset and persistence of depression are multifactorial. Key factors such as socioeconomic disadvantages, low educational attainment, unemployment, and adverse neighborhood conditions consistently predict an increased risk of depressive symptoms [4,5]. In Mexico, the ENSANUT [3] and other population-based studies documented a substantial burden of depressive symptoms that varies by age, sex, urban/rural residence, and socioeconomic status, highlighting significant gaps in detection and treatment coverage. Importantly, these social determinants operate directly and indirectly by modulating biological susceptibility [6,7].

Drawing on established pathophysiological frameworks—including the monoamine, neuroendocrine, neurotrophic, and neuroinflammatory theories—numerous studies have identified candidate genes implicated in depression [8,9]. To date, molecular pathway analyses have identified at least 11 common genes harboring specific variants significantly associated with major depressive disorder (MDD). Frequently investigated genes span multiple biological pathways, including serotonergic (e.g., solute carrier family 6 member 4 (*SLC6A4*), 5-hydroxytryptamine receptor 1A (*5-HT1A*) receptor gene, and tryptophan hydroxylase (*TPH*)), dopaminergic (e.g., dopamine transporter 1 (*DAT1*), dopamine receptor D4 (*DRD4*), and catechol-O-methyltransferase (*COMT*)), vascular and metabolic (e.g., apolipoprotein E (*APOE*), angiotensin-converting enzyme (*ACE*), and methylenetetrahydrofolate reductase (*MTHFR*)), glutamatergic (e.g., D-amino acid oxidase activator (*DAOA*)), and neurotrophic (e.g., brain-derived neurotrophic factor (*BDNF*)) systems [10].

The C-1019G (rs6295) polymorphism in the (*5-HT1A*) receptor gene modifies transcriptional regulation of the *5-HT1A* receptor and has been linked, across mechanistic, neuroimaging, and clinical studies, to altered amygdala reactivity and mood phenotypes [11]. The C-1019G polymorphism, which leads to increased *5-HT1A* autoreceptor expression, has been associated with depression risk, symptom severity, antidepressant response, and suicide, while heterogeneity has been noted across populations and study designs [12,13,14,15].

Bridging population-level social determinants with molecular variation offers a promising path to elucidate the mechanisms underlying vulnerability and resilience. This study aimed to integrate sociodemographic variables, clinical characteristics, and targeted genotyping of the C-1019G polymorphism to identify risk factors associated with depressive symptoms in a Mexican cohort.

## 2. Materials and Methods

### 2.1. Participants

All participants were recruited from the central region of Veracruz State, Mexico, encompassing Orizaba, Zapoapan, Tuxpanguillo, and Ixtaczoquitlán. Participant recruitment took place between 2020 and 2023 through structured interviews conducted at two sites: the Psychology Department of Sanitary Jurisdiction No. 7 Health Center (located in Zapoapan, Ixtaczoquitlán municipality) and the Orizaba Municipal DIF Clinic.

The surveys provided data on participants’ gender, age, marital status, educational attainment, and health status. Eligible participants included Mexican adults aged ≥ 18 years of both sexes with documented local ancestry (defined as having parents and grandparents born within the same community) who provided a venous blood sample. Exclusion criteria comprised (i) individuals who had family relationships outside the local population; (ii) pre-existing chronic conditions, such as renal, hepatic, pulmonary, or cardiac diseases and severe allergies; and (iii) pregnancy or lactation. Notably, individuals diagnosed with diabetes mellitus (DM) or arterial hypertension (AH) were intentionally retained in the study cohort despite these chronic conditions.

### 2.2. Beck Depression Inventory II (BDI-II)

The Beck Depression Inventory II (BDI-II) serves as a validated screening tool for assessing depressive symptomatology in adult populations. This 21-item self-report questionnaire quantifies depression severity based on symptoms experienced during the 2 weeks preceding administration. The total scores range from 0 to 63, with established clinical thresholds: ≤13 indicating no depressive symptoms, 14–19 suggesting mild depressive symptoms, 20–28 reflecting moderate depressive symptoms, and 29–63 signifying severe depressive symptoms [16]. A psychometric evaluation in a Mexican–American older adult cohort confirmed the instrument’s reliability (Cronbach’s α = 0.80) and validity [17]. All interviews and questionnaire assessments were conducted by trained mental health professionals. The complete BDI-II questionnaires were reviewed during the interview process to assess response consistency and completeness. Participants whose responses were considered unreliable or inconsistent, based on the clinical judgment of the evaluating professionals, were excluded from the study.

### 2.3. Study Design

Sociodemographic characteristics were categorized and analyzed in relation to BDI-II scores. For the case–control analysis, participants were divided into two groups based on established BDI-II thresholds: a control group comprising individuals with scores ≤ 13 (*n* = 202) and a depressive symptoms group comprising those with scores ≥ 14 (*n* = 55).

### 2.4. Genotyping

DNA was extracted from blood leukocytes with a Quick-DNA Kit (Zymo Research, Irvine, CA, USA). The C-1019G polymorphism was genotyped via polymerase chain reaction–restriction fragment length polymorphism (PCR-RFLP), following the methodology described by Rothe et al. [18], with minor modifications described by Hernández-Mixteco et al. [12]. Briefly, a 172-base pair (bp) fragment was amplified via PCR using the following primers (one modified): forward primer (F-mod): 5′-AGGGAGTAAGGCTGGACTGT-3′; reverse primer (R): 5′-GGAAGAAGACCGAGTGTGTCAT-3′. According to Kato et al. [19], the G allele resulted in an undigested 172 bp PCR product, while the C allele generated two fragments of 17 bp and 155 bp.

### 2.5. Statistical Analyses

The normality of the data was evaluated using the Shapiro–Wilk test, and the homogeneity of variances was assessed using the Bartlett test. To determine whether the data were in Hardy–Weinberg equilibrium, the χ^2^ test was used. The following tests were employed to compare groups: the Mann–Whitney U test for two groups and the Kruskal–Wallis test for three or more groups. The chi-square (χ^2^) test was used to examine the genotypes by genotypic distribution (G/G, C/G, and C/C) and allelic frequency (G and C) and under the recessive model (G/G + C/G vs. C/C) for the C-1019G polymorphism. Logistic regression was employed to evaluate the risk. The data are displayed as frequencies (with percentages) or means ± standard deviations for normally distributed variables and as medians with interquartile ranges for non-normally distributed variables. Statistical power was calculated using Pearson’s chi-square test to determine whether the sample size was sufficient to detect differences between case and control groups, sexes (male vs. female), and genotypes (G/G, C/G, and C/C). Statistical significance was defined as a *p*-value < 0.05. All analyses were performed using Stata 17.0 software (StataCorp LLC, College Station, TX, USA).

## 3. Results

### 3.1. Demographic and Clinical Characteristics of the Study Population

This study included 257 participants. A statistical power analysis indicated 100% power, demonstrating that this sample size was sufficient to detect differences between groups and was adequate for genetic analyses. A total of 85 participants (33.1%) were male, and 172 (66.9%) were female. The mean age was 37.14 (±20.67) years. Over half of the participants (56%) were under 40 years of age. Demographic analysis indicated that 60.3% were unmarried, and 56.1% reported high school as their highest level of educational attainment. In terms of health status, 146 participants (56.8%) were classified as healthy; 57 (22.2%) had DM; 29 (11.3%) had AH; 17 (6.6%) had both conditions (DM plus AH); and 8 (3.1%) reported other chronic conditions, including gastrointestinal, musculoskeletal, or thyroid disorders (Table 1).

### 3.2. Association of Demographic and Clinical Characteristics with Depressive Symptoms

The mean depressive symptom score was calculated from the BDI-II scores. It was found that male participants had lower BDI-II scores than female participants (*p* = 0.0003; z = −3.64; statistical power = 99%). Additionally, adults aged under 40 years had a lower BDI-II score than adults aged 40 to 65 years (*p* = 0.005) and adults aged over 65 years (*p* < 0.0001) (*p* = 0.0001; df = 2; χ^2^ = 26.39). Unmarried participants reported lower BDI-II scores than married or widowed/divorced participants (*p* = 0.0001; df = 2; χ^2^ = 18.45), and participants with a high school education had lower BDI-II scores than those with no education (*p* < 0.001) or elementary school education (*p* < 0.001) (*p* = 0.0001; df = 4; χ^2^ = 31.763). Regarding health status, it was found that individuals with DM and AH as comorbidities had higher BDI-II scores than healthy subjects (*p* < 0.0001) and individuals with DM (*p* = 0.019) or AH (*p* = 0.021) (*p* = 0.0001; df = 3; χ^2^ = 34.27) (Table 1).

### 3.3. Risk Analysis Between Demographic and Clinical Characteristics with Depressive Symptoms

Based on cases (subjects with depressive symptoms) and controls (healthy subjects), no significant differences were found between marital status and educational level as risk factors for presenting depressive symptoms (*p* > 0.05). However, it was found that females were three times more likely to be in the group with depressive symptoms than males (*p* = 0.004; odds ratio (OR): 3.08; 95% confidence interval (CI): 1.42–6.65). Additionally, adults aged 40 to 65 were twice as likely to experience depressive symptoms as those under 40 (*p* = 0.026; OR: 2.19; 95% CI: 1.09–4.57). Additionally, adults over 65 were five times more likely to experience depressive symptoms than individuals under 40 (*p* < 0.001; OR: 5.71; 95% CI: 2.45–13.30). Finally, regarding health status, it was found that individuals with type 2 DM had twice the risk of being in the group with depressive symptoms compared with healthy subjects (*p* = 0.025; OR: 2.38; 95% CI: 1.11–5.11). Of note, the risk of depressive symptoms increased twelvefold when participants had DM and AH as comorbidities (*p* < 0.001; OR: 12.25; 95% CI: 4.05–37.01).

### 3.4. Genotypic Distribution of C-1019G Polymorphism in the 5-HT1A Receptor Gene

The genotype distribution was as follows: G/G (*n* = 33, 12.84%), C/G (*n* = 134, 52.14%), and C/C (*n* = 90, 35.02%) (Table 2). The genotype frequencies were consistent with the Hardy–Weinberg equilibrium (*p* = 0.275, χ^2^ = 2.58).

### 3.5. Analysis of Genotype Frequencies and Recessive Model of the C-1019G Polymorphism in Control and Depressive Symptom Groups

No significant differences were observed in the distributions of the C/C, C/G, and G/G genotypes of the C-1019G polymorphism between the group with depressive symptoms and the control group in the overall study population (*p* = 0.397). Similarly, no significant differences were observed in the recessive model (G/G + C/G vs. C/C) analysis (*p* = 0.174; statistical power = 99.7%). In the sex-stratified analysis, no significant differences were observed in females for either genotype frequencies (*p* = 0.645; statistical power = 97.2%) or the recessive model (*p* = 0.705). However, in males, the C/C genotype was observed exclusively in the control group (*p* = 0.010), and this difference remained significant under the recessive model (*p* = 0.019; statistical power = 100%). Conversely, no significant association was observed between the G/G genotype and depressive symptoms (*p* > 0.05) (Table 2).

## 4. Discussion

Our results demonstrate a substantial relationship between demographic, clinical, and biological characteristics as determinants of depressive symptoms. First, we observed that male participants had a lower BDI-II score than female participants; additionally, female participants were three times more at risk of developing depressive symptoms than male participants. The sex difference between male and female patients can be explained by several biological, psychological, and social factors. MDD occurs approximately twice as often in females as in males [20]. Global estimates further support this pattern, reporting 201.27 million cases of depression in females compared with 131.14 million in males in 2021 [1]. This female predominance is observed from early adulthood through to old age, with higher prevalence and incidence rates reported in females across the lifespan [21,22]. From a biological perspective, sex differences have been partially attributed to genetic and hormonal factors [21]. The heritability of depression is estimated at 30–40%, with some evidence suggesting a stronger genetic vulnerability in females than in males [23]. Additionally, the activational effects of sex hormones beginning at puberty play a crucial role; hormonal fluctuations during puberty and other reproductive transitions are associated with an increased risk of depression in females, particularly in those with early pubertal timing or more advanced pubertal stages [21]. Of note, fluctuations in reproductive hormones may alter GABAergic regulation of the hypothalamic–pituitary–adrenal (HPA) axis, further contributing to emotional vulnerability in females [21,24]. However, biological explanations alone are insufficient. Psychosocial and cultural factors also play a significant role. Females are disproportionately exposed to stressors such as gender-based violence, including coercive, sexual, and severe physical partner violence, which are strongly associated with depression risk [25]. Structural inequalities, including limited access to economic resources and persistent gender discrimination, further exacerbate this vulnerability [25]. Despite our results, depressive symptoms in males should not be overlooked. Several studies have examined sex differences in the close relationship between depression and suicide. Ilić et al. [26] found that suicide mortality was higher among males than females across all age groups in the Serbian population. Furthermore, the WHO reports that the global suicide rate is 2.2 times higher in men than in women [27]. Moreover, Rice and Sher [28] found that social distancing and substance use can significantly increase the risk of suicide among adolescent males compared with their female peers.

We found that older subjects had higher BDI-II scores and a greater risk of experiencing depressive symptoms. These results are consistent with previous findings. Yang et al. [29] calculated that the risk of depression was higher in individuals aged 45 to 49 years than in those in their mid–late 20s. Panza et al. [30] found that in elderly individuals, depression was the second most common psychiatric disorder, and Hu et al. [31] calculated that the global prevalence of depression among elderly individuals was as high as 28.8%. Moreover, old age is associated with various health problems, such as chronic pain, Parkinson’s disease, DM, heart disease, stroke, chronic physical conditions, and other high-risk illnesses, which can affect the mental state of older adults and are highly correlated with geriatric depression [1,31,32]. These findings contrast with those of Kessler et al. [33], who observed that recent and lifetime major depressive episodes were significantly less frequent among U.S. adults aged 65 and older than among their younger counterparts. Additionally, they noted that the prevalence and severity of mental disorders generally declined with age.

The results show that unmarried participants had fewer symptoms of depression than married or widowed/divorced participants. This result contrasts with Buckman et al. [34], who evaluated the improvement of depression in adults under treatment, finding that unmarried participants had a worse prognosis of depression remission than married ones. Furthermore, two reviews reported better prognoses of depression for married individuals [35,36].

Regarding our results on educational level and depressive symptoms, we found that participants with a high school education had lower BDI-II scores than those with no formal education. This finding is supported by research that has established a strong correlation between educational attainment and cognitive function. Educational level not only impacts an individual’s susceptibility to depression but also influences their spouses and parents [37].

In this study, it was found that the co-occurrence of DM and AH increased the risk of depressive symptoms. This finding aligns with that of previous research, such as Read et al. [38], who reported that individuals with multimorbidity had twice the risk of developing a depressive disorder compared to those with a single chronic condition and nearly three times the risk compared to those without any chronic physical illness. The pathway from multimorbidity to depression may be mediated by factors such as increased symptom burden, functional disability, reduced quality of life, chronic pain, illness perceptions, and coping strategies [39,40,41]. Furthermore, metabolic and immune–inflammatory dysregulation, which are prevalent in many chronic diseases, have also been linked to depression [38,42].

Regarding biological factors, we found that the C/C genotype of the C-1019G polymorphism was present exclusively in males without depressive symptoms (the control group). These findings are consistent with those of previous studies. Takekita et al. [43] showed that carriers of the C allele of the C-1019G polymorphism experienced significantly greater improvement in depressive symptoms than those carrying the G allele. However, the results of other studies contrast with our findings. For example, Pawlowski et al. [14] reported that adult patients with chronic hepatitis C virus infection who carried the C/C genotype exhibited the highest baseline depression scores and the greatest increase in depressive symptoms during interferon treatment in a Polish cohort. Additionally, Galvao-de Almeida et al. [44] demonstrated an association between the C/C genotype of the C-1019G polymorphism and a clinical diagnosis of depression.

No association between the G/G genotype and depressive symptoms was found. This contrasts with previous studies that identified the G/G genotype as a risk factor for depression [45,46]. Moreover, Kraus et al. [47] reported that homozygosity for the G allele predisposed patients to depression. Similarly, Cozzolongo et al. [48] observed that patients carrying the G allele experienced greater depressive symptoms before and during interferon treatment, while Hernández-Díaz et al. [49] found that carriers of the G/G genotype exhibited an increased risk of suicidal behavior in the general population.

The genotype frequencies observed in our study differ significantly from those reported in populations from Tabasco (Mexico), China, India, Austria, Belgium, France, Italy, Israel, South Korea, Poland, Turkey, and Iran (*p* < 0.05) (Table 3). These results indicate that the genotype frequencies in our study population differ from those in other populations previously linked to depressive symptoms. Conversely, the allele frequencies are similar to those reported in populations from Tabasco (Mexico), India, Austria, Belgium, France, Italy, Israel, and Turkey (Table 3). Research on the C-1019G (rs6295) polymorphism of the *5-HT1A* receptor gene in Latin America remains scarce, as most published studies have focused on European and Asian populations. Consequently, this study in a Mexican cohort represents one of the first to specifically analyze this polymorphism in the region.

The observed differences in genotype and allele frequencies between populations are unsurprising and may reflect distinct ancestries, demographic history, and evolutionary processes such as genetic drift, migration, and natural selection. Human populations exhibit distinct allele frequency distributions that have been shaped by historical geographic isolation, founder effects, and admixture. These factors are particularly relevant in the Mexican population, which is characterized by a complex genetic background resulting from varying proportions of Native American, European, and African ancestries. Consequently, differences in genetic structure between populations may account for the variability observed in the distribution of the rs6295 polymorphism across studies [60].

This study has several limitations. First, our findings may vary across populations, underscoring the need for future research that integrates biological, psychological, and social dimensions to better understand the mechanisms underlying depressive symptoms. Second, although we adjusted for various potential confounders, residual confounding cannot be entirely ruled out. Third, it is important to note that the BDI-II was used to assess depressive symptoms rather than to establish a clinical diagnosis of depression. Finally, although our sample size provided adequate statistical power for the analyses, larger samples are generally preferred in genetic association studies to improve the precision of effect estimates and enhance the robustness of the findings. Therefore, future studies with larger cohorts and more diverse genetic backgrounds are needed to validate our results.

## 5. Conclusions

This preliminary study highlights the multifactorial nature of depression, demonstrating that social and biological determinants significantly contribute to depressive symptoms in a rural population, a group historically underrepresented in the literature. In this Mexican cohort, female sex, older age, and the presence of comorbidities were associated with an increased risk of depressive symptoms. The analysis suggested that the C/C genotype may confer a protective effect against depressive symptoms in males; however, given the small sample size of male participants, this finding should be interpreted with caution. Finally, the genotypic distribution of this study population differed from that of European and Asian populations.

## Figures and Tables

**Table 1:** Characterization of the study population.

Characterization *N* = 257	*N* (%)	BDI-II * Score (Mean ± Standard Deviation)	*p*-Value
*Sex* Male Female	85 (33.1) 172 (66.9)	7.27 ± 7.31 10.51 ± 8.44	0.0003 ^
*Age in years* <40 40 to 65 >65	144 (56.0) 81 (31.5) 32 (12.5)	7.45 ± 7.08 ^ab^ 10.92 ± 7.80 ^a^ 14.65 ± 10.79 ^b^	0.0001 ^+^
*Marital status* Unmarried Married Widowed/divorced	155 (60.3) 87 (33.9) 15 (5.8)	7.87 ± 7.37 ^a^ 11.80 ± 8.74 ^a^ 11.93 ± 9.96	0.0001 ^+^
*Level of education* No education Elementary school Middle school High school Bachelor’s school	14 (5.4) 67 (26.1) 19 (7.3) 144 (56.1) 13 (5.1)	16.57 ± 12.95 ^a^ 12.40 ± 8.38 ^b^ 9.63 ± 6.91 7.09 ± 6.36 ^ab^ 12.30 ± 11.58	<0.0001 ^+^
*Health status* Healthy Diabetes mellitus (DM) Arterial hypertension (AH) DM and AH Others **	146 (56.8) 57 (22.2) 29 (11.3) 17 (6.6) 8 (3.1)	7.26 ± 6.87 ^ac^ 11.43 ± 8.88 ^ad^ 10.79 ± 7.72 ^b^ 18.11 ± 9.57 ^bcd^ 11.75 ± 9.22	0.0001 ^+^

* Beck Depression Inventory II; ** gastrointestinal, musculoskeletal, or thyroid disorders; ^ Mann–Whitney U test; ^+^ Kruskal–Wallis test; ^a–d^ there is a statistically significant difference between identical letters.

**Table 2:** Genotype and recessive model frequencies for the C-1019G (rs6295) polymorphism of the *5-HT1A* receptor gene between control and depressive symptom groups.

	Genotype				Recessive Model		
Entire population (*N* = 257)	G/G	C/G	C/C	*p*	G/G + G/C	C/C	*p*
Controls (*N* = 202)	25	102	75	0.397 *	127	75	0.174 *
Individuals with depressive symptoms (*N* = 55)	8	32	15		40	15	
*Females (N = 172*)							
Controls (*N* = 126)	16	65	45	0.645 *	81	45	0.705 *
Individuals with depressive symptoms (*N* = 46)	4	27	15		31	15	
*Males (N = 85)*							
Controls (*N* = 76)	9	37	30	0.010 *	46	30	0.019 *
Individuals with depressive symptoms (*N* = 9)	4	5	0		9	0	

* chi-square test.

**Table 3:** Genotypic and allelic frequencies of the rs6295 polymorphism of the *5-HT1A* receptor gene in different populations with depressive symptoms.

Region/Country	C/C *N* (%)	C/G *N* (%)	G/G *N* (%)	Chi^2^ *p*-Value	Allelic Frequency C Allele G Allele		Chi^2^ *p*-Value	Reference
Veracruz, México	90 (35.02)	134 (52.14)	33 (12.84)		0.611	0.389		This study
Tabasco, México	152 (36.54)	183 (43.99)	81 (19.47)	6.47, *p* = 0.039	0.622	0.378	0.03, *p* = 0.873	[50]
China	73 (6.40)	425 (37.24)	643 (56.36)	241.14, *p* < 0.001	0.25	0.75	26.58, *p* < 0.001	[51]
China	361 (62.35)	185 (31.95)	33 (5.70)	55.15, *p* < 0.001	0.783	0.217	6.93, *p* = 0.008	[52]
India	61 (58.0)	32 (31.0)	11 (11)	17.55, *p* < 0.001	0.74	0.26	3.8, *p* = 0.051	[53]
Multicenter study (Austria, Belgium, France, Italy, Israel)	57 (23.55)	120 (49.6)	65 (26.85)	18.19, *p* < 0.001	0.483	0.517	3.31, *p* = 0.069	[54]
Italy	28 (25.22)	51 (45.96)	32 (28.82)	14.13, *p* = 0.001	0.481	0.519	3.41, *p* = 0.065	[55]
Republic of Korea	12 (3.22)	127 (34.13)	233 (62.65)	195.73, *p* < 0.001	0.203	0.797	34.49, *p* < 0.001	[56]
Austria	18 (22.22)	41 (50.61)	22 (27.17)	10.95, *p* = 0.004	0.475	0.525	3.73, *p* = 0.054	[11]
Poland	92 (20.04)	248 (54.04)	119 (25.92)	27.94, *p* < 0.001	0.47	0.53	4.0, *p* = 0.045	[57]
Turkey	137 (33.82)	179 (44.20)	89 (21.98)	9.28, *p* = 0.01	0.56	0.44	0.54, *p* = 0.464	[58]
Iran	40 (10.13)	175 (44.30)	180 (45.57)	101.46, *p* < 0.001	0.322	0.678	16.71, *p* < 0.001	[59]

## Data Availability

The data presented in this study are available upon request from the corresponding author due to participant privacy.

## Abbreviations

The following abbreviations are used in this manuscript:

*5-HT1A*	Serotonin 1A receptor gene
AH	Arterial hypertension
BDI-II	Beck Depression Inventory II
DM	Diabetes mellitus

## References

[1] Chen X.-D., Li F., Zuo H., Zhu F. (2025). Trends in Prevalent Cases and Disability-Adjusted Life-Years of Depressive Disorders Worldwide: Findings from the Global Burden of Disease Study from 1990 to 2021. Depress. Anxiety.

[2] Errazuriz A., Avello-Vega D., Ramirez-Mahaluf J.P., Torres R., Crossley N.A., Undurraga E.A., Jones P.B. (2023). Prevalence of depressive disorder in the adult population of Latin America: A systematic review and meta-analysis. Lancet Reg. Health Am..

[3] Vazquez-Salas A., Hubert C., Portillo-Romero A., Valdez-Santiago R., Barrientos-Gutierrez T., Villalobos A. (2023). Sintomatología depresiva en adolescentes y adultos mexicanos: Ensanut 2022. Salud Publica Mex..

[4] Kirkbride J.B., Anglin D.M., Colman I., Dykxhoorn J., Jones P.B., Patalay P., Pitman A., Soneson E., Steare T., Wright T. (2024). The social determinants of mental health and disorder: Evidence, prevention and recommendations. World Psychiatry.

[5] Jespersen A., Madden R.A., Whalley H.C., Reynolds R.M., Lawrie S.M., McIntosh A.M., Iveson M.H. (2025). Socioeconomic status and depression—A systematic review. Epidemiol. Rev..

[6] Cerecero-Garcia D., Macias-Gonzalez F., Aramburo-Muro T., Bautista-Arredondo S. (2020). Depressive symptoms and coverage of diagnosis and treatment of depression in Mexican population. Salud Publica Mex..

[7] Garcia-Perez A., Gonzalez-Aragon Pineda A.E., Sandoval-Bonilla B.A., Cruz-Hervert L.P. (2022). Prevalence and factors associated with the depressive symptoms in rural and urban Mexican older adults: Evidence from the Mexican Health and Aging Study 2018. Salud Publica Mex..

[8] Belmaker R.H., Agam G. (2008). Major depressive disorder. N. Engl. J. Med..

[9] Malhi G.S., Mann J.J. (2018). Depression. Lancet.

[10] Suktas A., Ekalaksananan T., Aromseree S., Bumrungthai S., Songserm N., Pientong C. (2024). Genetic polymorphism involved in major depressive disorder: A systemic review and meta-analysis. BMC Psychiatry.

[11] Kautzky A., James G.M., Philippe C., Baldinger-Melich P., Kraus C., Kranz G.S., Vanicek T., Gryglewski G., Wadsak W., Mitterhauser M. (2017). The influence of the rs6295 gene polymorphism on serotonin-1A receptor distribution investigated with PET in patients with major depression applying machine learning. Transl. Psychiatry.

[12] Hernandez-Mixteco M., Valenzuela O.L., Balderas-Vazquez C.L., Castillo-Juarez P., Rivera-Gutierrez S., Garcia-Reyes R.L., Cornejo-Estudillo G., Soria-Herrera R.J., Leon-Juarez M., Helguera-Repetto A.C. (2025). The C/C Genotype of the C-1019G (rs6295) Polymorphism of the *5-HT1A* Receptor Gene Is Associated with Lower Susceptibility to Depressive Symptoms in a Rural Population in Mexico. Neurol. Int..

[13] Wu H.-N., Zhu S.-Y., Zhang L.-N., Shen B.-H., Xu L.-L. (2024). Association between 5-HTR1A gene C-1019G polymorphism and antidepressant response in patients with major depressive disorder: A meta-analysis. World J. Psychiatry.

[14] Pawlowski T., Malyszczak K., Pawlak D., Inglot M., Zalewska M., Grzywacz A., Radkowski M., Laskus T., Janocha-Litwin J., Frydecka D. (2023). HTR1A, TPH2, and 5-HTTLPR Polymorphisms and Their Impact on the Severity of Depressive Symptoms and on the Concentration of Tryptophan Catabolites during Hepatitis C Treatment with Pegylated Interferon-alpha2a and Oral Ribavirin (PEG-IFN-alpha2a/RBV). Cells.

[15] Albert P.R., Le Francois B., Vahid-Ansari F. (2019). Genetic, epigenetic and posttranscriptional mechanisms for treatment of major depression: The *5-HT1A* receptor gene as a paradigm. J. Psychiatry Neurosci..

[16] Segal D.L., Coolidge F.L., Cahill B.S., O’Riley A.A. (2008). Psychometric properties of the Beck Depression Inventory II (BDI-II) among community-dwelling older adults. Behav. Modif..

[17] Gatewood-Colwell G., Kaczmarek M., Ames M.H. (1989). Reliability and validity of the Beck Depression Inventory for a white and Mexican-American gerontic population. Psychol. Rep..

[18] Rothe C., Gutknecht L., Freitag C., Tauber R., Mossner R., Franke P., Fritze J., Wagner G., Peikert G., Wenda B. (2004). Association of a functional 1019C>G *5-HT1A* receptor gene polymorphism with panic disorder with agoraphobia. Int. J. Neuropsychopharmacol..

[19] Kato M., Fukuda T., Wakeno M., Okugawa G., Takekita Y., Watanabe S., Yamashita M., Hosoi Y., Azuma J., Kinoshita T. (2009). Effect of *5-HT1A* gene polymorphisms on antidepressant response in major depressive disorder. Am. J. Med. Genet. Part B Neuropsychiatr. Genet..

[20] Otte C., Gold S.M., Penninx B.W., Pariante C.M., Etkin A., Fava M., Mohr D.C., Schatzberg A.F. (2016). Major depressive disorder. Nat. Rev. Dis. Primers.

[21] Kuehner C. (2017). Why is depression more common among women than among men?. Lancet Psychiatry.

[22] Aziz R., Steffens D.C. (2013). What are the causes of late-life depression?. Psychiatr. Clin. N. Am..

[23] Sullivan P.F., Neale M.C., Kendler K.S. (2000). Genetic epidemiology of major depression: Review and meta-analysis. Am. J. Psychiatry.

[24] Graber J.A. (2013). Pubertal timing and the development of psychopathology in adolescence and beyond. Horm. Behav..

[25] Howard L.M., Trevillion K., Agnew-Davies R. (2010). Domestic violence and mental health. Int. Rev. Psychiatry.

[26] Ilic M., Ilic I. (2016). Suicide in Serbia. J. Affect. Disord..

[27] World Health Organization (WHO) (2024). Suicide Worldwide in 2021: Global Health Estimates. https://www.who.int/publications/i/item/9789240110069.

[28] Rice T., Sher L. (2021). The men’s mental health perspective on adolescent suicide in the COVID-19 era. Acta Neuropsychiatr..

[29] Yang J.S., Zhang L.Y., Yang C.H., Li X.Y., Li Z.Q. (2024). Global, Regional, and National Epidemiology of Depression in Working-Age Individuals, 1990–2019. Depress. Anxiety.

[30] Panza F., Frisardi V., Capurso C., D’Introno A., Colacicco A.M., Imbimbo B.P., Santamato A., Vendemiale G., Seripa D., Pilotto A. (2010). Late-life depression, mild cognitive impairment, and dementia: Possible continuum?. Am. J. Geriatr. Psychiatry.

[31] Hu T., Zhao X., Wu M., Li Z., Luo L., Yang C., Yang F. (2022). Prevalence of depression in older adults: A systematic review and meta-analysis. Psychiatry Res..

[32] Chang-Quan H., Xue-Mei Z., Bi-Rong D., Zhen-Chan L., Ji-Rong Y., Qing-Xiu L. (2010). Health status and risk for depression among the elderly: A meta-analysis of published literature. Age Ageing.

[33] Kessler R.C., Birnbaum H., Bromet E., Hwang I., Sampson N., Shahly V. (2010). Age differences in major depression: Results from the National Comorbidity Survey Replication (NCS-R). Psychol. Med..

[34] Buckman J.E.J., Saunders R., Stott J., Arundell L.L., O’Driscoll C., Davies M.R., Eley T.C., Hollon S.D., Kendrick T., Ambler G. (2021). Role of age, gender and marital status in prognosis for adults with depression: An individual patient data meta-analysis. Epidemiol. Psychiatr. Sci..

[35] Carter G.C., Cantrell R.A., Victoria Z., Haynes V.S., Phillips G., Alatorre C.I., Goetz I., Paczkowski R., Marangell L.B. (2012). Comprehensive review of factors implicated in the heterogeneity of response in depression. Depress. Anxiety.

[36] Sockol L.E. (2018). A systematic review and meta-analysis of interpersonal psychotherapy for perinatal women. J. Affect. Disord..

[37] Lee J. (2011). Pathways from education to depression. J. Cross Cult. Gerontol..

[38] Read J.R., Sharpe L., Modini M., Dear B.F. (2017). Multimorbidity and depression: A systematic review and meta-analysis. J. Affect. Disord..

[39] Bair M.J., Robinson R.L., Katon W., Kroenke K. (2003). Depression and pain comorbidity: A literature review. Arch. Intern. Med..

[40] Katon W.J. (2003). Clinical and health services relationships between major depression, depressive symptoms, and general medical illness. Biol. Psychiatry.

[41] Ziarko M., Mojs E., Piasecki B., Samborski W. (2014). The mediating role of dysfunctional coping in the relationship between beliefs about the disease and the level of depression in patients with rheumatoid arthritis. Sci. World J..

[42] Penninx B.W. (2017). Depression and cardiovascular disease: Epidemiological evidence on their linking mechanisms. Neurosci. Biobehav. Rev..

[43] Takekita Y., Fabbri C., Kato M., Koshikawa Y., Tajika A., Kinoshita T., Serretti A. (2016). HTR1A Polymorphisms and Clinical Efficacy of Antipsychotic Drug Treatment in Schizophrenia: A Meta-Analysis. Int. J. Neuropsychopharmacol..

[44] Galvao-de Almeida A., Quarantini L.C., Tartaglioni A.G., Lyra A.C., Parise C.L., Parana R., de Oliveira I.R., Miranda-Scippa A., Guindalini C. (2014). Serotonin-1A receptor CC genotype is associated with persistent depression related to interferon-alpha in hepatitis C patients. Gen. Hosp. Psychiatry.

[45] Parsey R.V., Olvet D.M., Oquendo M.A., Huang Y.Y., Ogden R.T., Mann J.J. (2006). Higher *5-HT1A* receptor binding potential during a major depressive episode predicts poor treatment response: Preliminary data from a naturalistic study. Neuropsychopharmacology.

[46] Kim H.K., Kim S.J., Lee Y.J., Lee H.J., Kang S.G., Choi J.E., Yun K.W., Lim W.J. (2011). Influence of the interaction between the serotonin 1A receptor C-1019G polymorphism and negative life stressors on the development of depression. Neuropsychobiology.

[47] Kraus M.R., Al-Taie O., Schafer A., Pfersdorff M., Lesch K.P., Scheurlen M. (2007). Serotonin-1A receptor gene HTR1A variation predicts interferon-induced depression in chronic hepatitis C. Gastroenterology.

[48] Cozzolongo R., Porcelli P., Cariola F., Giannuzzi V., Lanzilotta E., Gentile M., Sonnante G., Leandro G. (2015). Serotonin gene polymorphisms and lifetime mood disorders in predicting interferon-induced depression in chronic hepatitis C. J. Affect. Disord..

[49] Hernandez-Diaz Y., Tovilla-Zarate C.A., Castillo-Avila R.G., Juarez-Rojop I.E., Genis-Mendoza A.D., Lopez-Narvaez M.L., Villar-Juarez G.E., Gonzalez-Castro T.B. (2023). Association between the HTR1A rs6295 gene polymorphism and suicidal behavior: An updated meta-analysis. Eur. Arch. Psychiatry Clin. Neurosci..

[50] Gonzalez-Castro T.B., Tovilla-Zarate C.A., Juarez-Rojop I., Pool Garcia S., Genis A., Nicolini H., Lopez Narvaez L. (2013). Association of 5HTR1A gene variants with suicidal behavior: Case-control study and updated meta-analysis. J. Psychiatr. Res..

[51] Huang J.-H., Chang H.-A., Fang W.-H., Ho P.-S., Liu Y.-P., Wan F.-J., Tzeng N.-S., Shyu J.-F., Chang C.-C. (2018). Serotonin receptor 1A promoter polymorphism, rs6295, modulates human anxiety levels via altering parasympathetic nervous activity. Acta Psychiatr. Scand..

[52] Liu J., Gong P., Zhou X. (2014). The association between romantic relationship status and 5-HT1A gene in young adults. Sci. Rep..

[53] Tanwar S., Mattoo B., Kumar U., Dada R., Bhatia R. (2021). Does human serotonin-1A receptor polymorphism (rs6295) code for pain and associated symptoms in fibromyalgia syndrome?. Reumatismo.

[54] Hofer P., Schosser A., Calati R., Serretti A., Massat I., Kocabas N.A., Konstantinidis A., Mendlewicz J., Souery D., Zohar J. (2016). The impact of serotonin receptor 1A and 2A gene polymorphisms and interactions on suicide attempt and suicide risk in depressed patients with insufficient response to treatment--a European multicentre study. Int. Clin. Psychopharmacol..

[55] Pompili M., Gentile G., Scassellati C., Bonvicini C., Innamorati M., Erbuto D., Montebovi F., Ducci G., Forte A., De Pisa E. (2017). Genetic association analysis of serotonin and signal transduction pathways in suicide attempters from an Italian sample of psychiatric patients. Neurosci. Lett..

[56] Choi H.Y., Kim G.E., Kong K.A., Lee Y.J., Lim W.J., Park S.H., Ha S.H., Kim S.I. (2018). Psychological and genetic risk factors associated with suicidal behavior in Korean patients with mood disorders. J. Affect. Disord..

[57] Huminska-Lisowska K., Chmielowiec J., Chmielowiec K., Stronska A., Cieszczyk P., Spieszny M., Masiak J., Lachowicz M., Surala O., Grzywacz A. (2024). Association between polymorphism rs6295 of HTR1A serotonin receptor gene and personality traits among athletes of combat sport. Biol. Sport.

[58] Ates O., Karakus N., Sezer S., Bozkurt N. (2013). Genetic association of 5-HT1A and 5-HT1B gene polymorphisms with migraine in a Turkish population. J. Neurol. Sci..

[59] Alizadeh N., Nosrat N., Jahani Z., Ahmadiani A., Asadi S., Shams J. (2019). Association of HTR1A gene polymorphisms with obsessive-compulsive disorder and its treatment response: The influence of sex and clinical characteristics. Int. J. Neurosci..

[60] Adhikari K., Chacon-Duque J.C., Mendoza-Revilla J., Fuentes-Guajardo M., Ruiz-Linares A. (2017). The Genetic Diversity of the Americas. Annu. Rev. Genom. Hum. Genet..

